# Electrophysiological and behavioral responses of fourth instar spotted lanternfly nymphs to hexanal

**DOI:** 10.17912/micropub.biology.001861

**Published:** 2025-11-03

**Authors:** Raghav Bharadwaj, Qi Xue, Gale E. Ridge, Claire E. Rutledge, Hany K. M. Dweck

**Affiliations:** 1 Department of Entomology, Connecticut Agricultural Experiment Station, New Haven, Connecticut, United States; 2 North Haven High School, North Haven, CT, USA

## Abstract

The spotted lanternfly ( *Lycorma delicatula* ) is an invasive, polyphagous planthopper. We previously identified an olfactory organ on the labium in adults; here, we show that fourth instar nymphs possess a similar organ. This organ consists of two placoid sensilla, each housing two olfactory receptor neurons: one tuned to hexanal and the other to methyl salicylate. We also found that 10 µl of pure hexanal elicits strong attraction in nymphs. These results highlight the olfactory capabilities of SLF nymphs and identify hexanal as a potential attractant that could aid in monitoring and management of this insect.

**Figure 1. Responses of fourth instar nymphs to hexanal.**
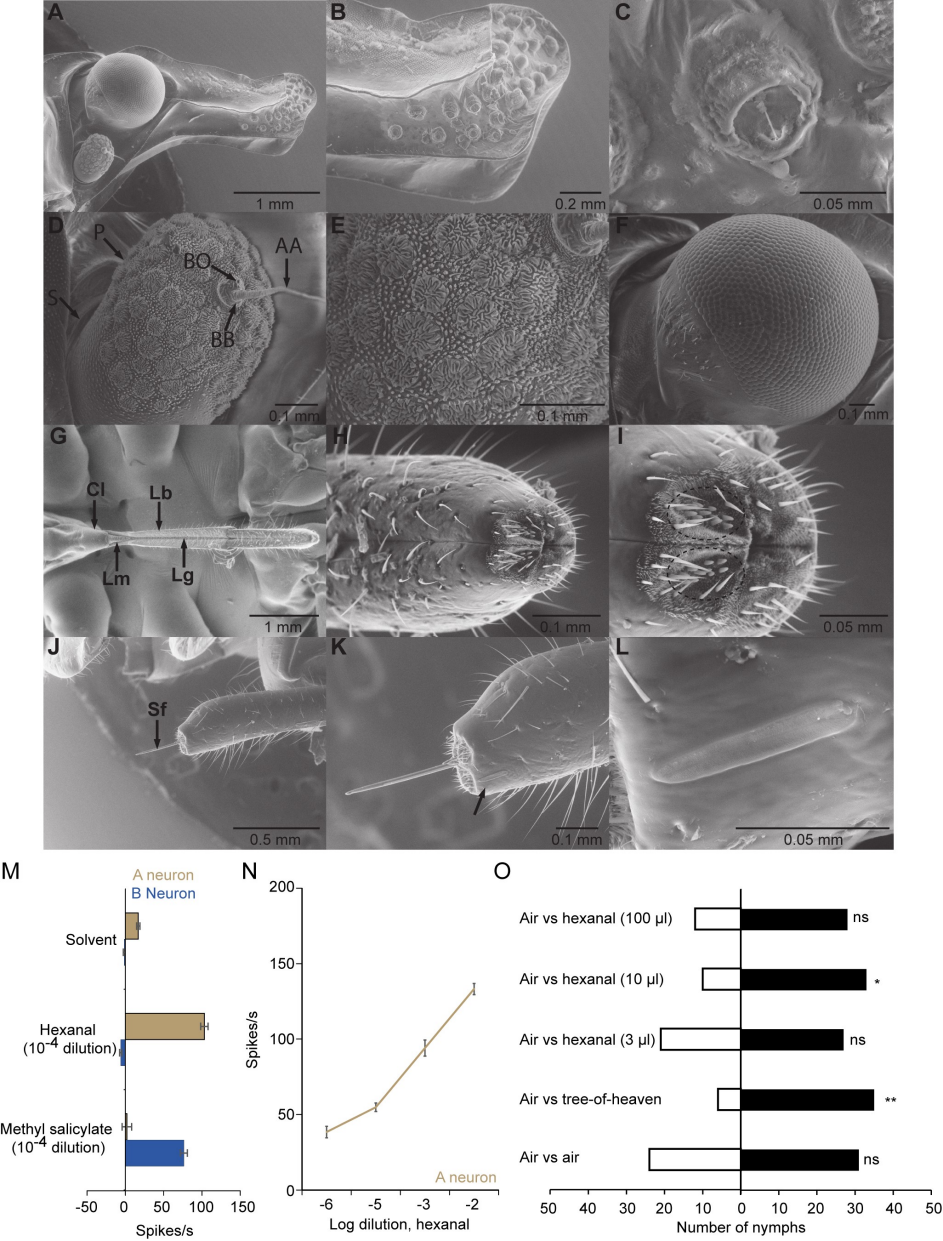
(A) Lateral view of the head showing the antenna, eye, and anterior region of frons.(B) Enlarged view of the anterior region of the frons and the sensory pits.(C) Enlarged view of a single sensory pit showing a horizontal hair and a raised rim on side opposite the hair.(D) General view of the antenna, which consists of scape (S), pedicel (P) and flagellum (F). The flagellum consists of a thread like apical arista (AA) and a basal bulb (BB) which possesses the internal Bourgoin sensory organ (BO). (E) Enlarged view of the antenna showing various sensory structures.(F) General view of the eye.(G) Anterior view of the mouthparts showing the clypeus (Cl), labrum (Lm), labium (Lb), and labial groove (Lg).(H) Enlarged view of the fourth labial segment.(I) Enlarged view of the tip of the fourth labial segment showing the apical sensory field and its sensory pegs (dashed circle) on each lobe of the labium.(J) Lateral view of the fourth labial segment showing the stylet fascicle (Sf).(K) Enlarged lateral view of the fourth labial segment showing the subapical sensory organ (indicated by arrow).(L) Enlarged view of the subapical sensory organ.(M) Responses of neurons housed in placoid sensilla to solvent control (hexane), hexanal, and methyl salicylate. Error bars are SEM. n = 5. (N) Responses of the A neurons housed in placoid sensilla to four doses of hexanal. Error bars are SEM. n = 5.(O) Behavioral responses of fourth instar nymphs to air versus air, air versus tree-of-heaven, and air versus different doses of hexanal. ns, not significant; *P < 0.05; **P < 0.01; Fisher’s exact test. n=40-55.

## Description

Adults and nymphs (four instar stages) of SLF pose a serious threat to agriculture, forestry, and urban landscapes (Urban and Leach, 2023). While research and control efforts have largely focused on adults (Urban and Leach, 2023), nymphs particularly fourth instars, which share similar host plant preferences with adults (Liu, 2019), remain understudied.

Here, we examined the head and mouthparts of fourth instar SLF nymphs using scanning electron microscopy to identify sensory structures and potential functions. On the head, we observed more than 20 sensory pits on each side of the anterior region of the frons ( Figure 1A, B). Each pit possessed a single horizontal hair opposite a raised rim ( Figure 1C ). Similar structures with the same location but variable hair morphology have been described in other hemipteran species (Bräunig et al., 2012). Based on their lateral position and asymmetric arrangement between the left and right sides, these hairs are likely involved in detecting wind direction.

On the antennae (composed of scape, pedicel, and flagellum), we found many various shaped plate organs on the pedicel, as well as several other sensory structures ( Figure 1D, E). Plate organs are commonly found in hemipterans. These are multi-porous and believed to serve an olfactory function (Bourgoin and Deiss, 1994; Stroiński et al., 2011; Wang et al., 2018).

On the labium, we observed both apical and subapical sensory organs on the fourth segment. The apical sensory organ consists of two fields, each with ~10 sensory pegs on the dorsal surface, along with additional sensory hairs ( Figure 1G –I). In adult SLF, some of these pegs are multi-porous and are proposed to serve an olfactory function, while others possess an apical pore and are thought to have a gustatory role (Brożek and Bourgoin, 2013; Hao et al., 2016). The subapical sensory organ is located laterally near the tip of the segment and contains two placoid sensilla ( Figure 1J –L).

**Placoid sensilla of the subapical labial sensor organ in fourth instar nymphs**

The presence of a subapical sensory organ on the fourth labial segment of fourth instar nymphs led us to test whether it serves an olfactory function. In adults, the two placoid sensilla of this organ are structurally identical and each has two olfactory receptor neurons (Dweck and Rutledge, 2024). One of these neurons responds selectively to a limited set of chemical compounds, with hexanal eliciting the strongest excitatory response while the second neuron responds to many compounds, among which methyl salicylate is the most potent activator.

To determine whether the same functional organization exists in the nymphs, we performed single sensillum recordings on their two placoid sensilla using a range of hexanal dilutions and a single dilution of methyl salicylate. Our recordings revealed that, as in adults, each sensillum contains two neurons ( Figure 1M ). The neuron with larger spikes exhibited a dose-dependent response to hexanal but was unresponsive to methyl salicylate ( Figure 1M, N)). Conversely, the neuron with smaller spikes responded to methyl salicylate and showed no response to hexanal at any test dilution ( Figure 1M ). These results indicate the functional identity of these two neurons is conserved between nymphs and adults.

**Hexanal elicits strong attraction in fourth instar nymphs**

We assessed the behavioral responses of fourth instar nymphs to hexanal, which had not been tested previously, using a Y-tube glass olfactometer. First, we tested clean air versus clean air and observed no preference for either arm ( Figure 1O ). Next, we tested the odor of tree of heaven, the most preferred host for SLF, and confirmed it elicited strong attraction ( Figure 1O ). Finally, we tested three doses of pure hexanal and found that 10 µl elicited strong attraction, while 3 µl and 100 µl elicited neutral responses ( Figure 1O ).

Previous studies have emphasized the role of methyl salicylate as a key semiochemical for both adult and nymph stages of SLF (Booth et al., 2025; Cooperband and Murman, 2024; Cooperband et al., 2019; Derstine et al., 2020). Our results extend this by identifying hexanal as an additional volatile capable of influencing SLF nymph behavior. Hexanal is a well-characterized green leaf volatile released by host plants in response to mechanical injury or herbivory, and its emission levels typically increase with the intensity of feeding damage and infestation pressure (Matsui and Engelberth, 2022; Scala et al., 2013; ul Hassan et al., 2015). Such a pattern makes hexanal an ecologically reliable cue that could signal the presence of conspecific activity or suitable feeding sites. Importantly, we found that SLF nymphs exhibit attraction to a specific dose of hexanal, suggesting that this compound may function not only as a host-derived cue but also as a potential aggregation signal that promotes conspecific clustering.

## Methods

**Fourth instar nymphs**

Fourth instar nymphs were collected daily from tree of heaven *Ailanthus altissima* (New Haven, CT) and transported to our research laboratories at The Connecticut Agricultural Experiment Station campus in New Haven, CT, in accordance with U.S. Department of Agriculture (USDA) permit 526-24-114- 68354. Fourth instar SLF nymphs used in the Y-tube behavioral experiments were selectively field collected from locations where there were no adults present, and the majority of the nymphs were in the fourth instar stage. In the laboratories, they were housed for one day in rearing cages (BugDorm-4M3030 Insect Rearing Cage) at 23°C and 40% relative humidity, with access to tree of heaven for feeding.

**Scanning electron microscopy images**

Scanning electron microscopy images were captured using a Hitachi Tabletop Microscope TM3030Plus from Hitachi High-Technologies Corporation, Tokyo, Japan. Three individuals were examined.

**Odorants**

Analytical standards of hexanal (catalog #18109) and methyl salicylate (catalog #76631) were purchased from Millipore Sigma. These odorants were dissolved in hexane (Millipore Sigma; catalog #139386).

**Single sensillum recordings**

Single sensillum recordings technique in SLF is described in detail in (Dweck, 2024).

**Y-tube olfactometer behavioral bioassays**

A Y-tube Pyrex glass olfactometer, custom-made at the Yale Sterling Chemistry Scientific Glassblowing Laboratory (New Haven, CT), was used to measure the behavioral responses of fourth instar SLF nymphs to different stimuli. The glass Y-tube was 5 cm in diameter, with a central main tube 23 cm long and two lateral arms 21 cm in length. Air from an air pump (WELCH Piston Air Compressor/Vacuum Pump: 0.033 hp, 1 Phase, 115V AC, 33 psi max continuous pressure) was passed through activated carbon filters, bubbled through water, and regulated with a flow meter (Analytical Research Systems, Gainesville, FL) before being split and directed into two 50-ml glass bottles. One bottle contained either the pure chemical or a leaf of the tree of heaven, while the other bottle was left empty as a control. Each bottle was connected to one of the lateral arms of the Y-tube via tubing (2.5 mm inner diameter). The airflow in each arm was maintained at 50 cm/s. Experiments were conducted between 0900 and 1600 hours under fluorescent red-light bulbs (60-Watt Equivalent) positioned in front of the Y-tube olfactometer to minimize visual cues and ensure that insect choices were guided primarily by olfactory stimuli. After every ten trials, a clean Y-tube olfactometer was used, and the stimulus was refreshed, and its side was switched.

For each trial, a single nymph was individually introduced into the central arm of the Y-tube. We recorded the initial choice of each nymph that walked into one of the lateral arms and remained there for at least 1 minute. If a nymph did not make a choice within 10 minutes of being released into the olfactometer, it was excluded from the analysis.

The doses of hexanal used in these experiments were 3, 10, and 100 µL of pure odorant, corresponding to 2.44 mg, 8.13 mg, and 81.3 mg, respectively. We were unable to test the 30 µL dose because, at the time it was scheduled for testing, the fourth instar nymphs had begun to molt into adults.

Each trial was conducted with a new individual, and the experiments were performed over several days.

**Statistical analysis**

The fisher exact test for each stimulus was calculated in GraphPad Prism 10.5.0 (774).
